# Latent *Toxoplasma gondii* Infection Does Not Modulate Immune Aging in a Cross-Sectional Working-Age Population Study

**DOI:** 10.3390/biom16010055

**Published:** 2025-12-30

**Authors:** Peter Bröde, Maren Claus, Stephan Getzmann, Klaus Golka, Jan G. Hengstler, Jörg Reinders, Edmund Wascher, Carsten Watzl, Patrick D. Gajewski

**Affiliations:** 1 Leibniz Research Centre for Working Environment and Human Factors at TU Dortmund (IfADo), Ardeystraße 67, D-44139 Dortmund, Germany; broede@ifado.de (P.B.); claus@ifado.de (M.C.); getzmann@ifado.de (S.G.); golka@ifado.de (K.G.); hengstler@ifado.de (J.G.H.); reinders@ifado.de (J.R.); wascher@ifado.de (E.W.); watzl@ifado.de (C.W.); 2 German Center for Mental Health (DZPG), Partner Site Bochum/Marburg, Massenbergstraße 9–13, D-44787 Bochum, Germany

**Keywords:** toxoplasmosis, immunosenescence, ageing, biomarker, IMMAX, Dortmund Vital Study

## Abstract

Latent, i.e., asymptomatic *Toxoplasma gondii* (*T. gondii*) infection might accelerate or modulate the aging process of cognitive and sensory functions involving pro-inflammatory immune responses. For evaluating a potential role of latent *T. gondii* infection in immunological aging, we determined *T. gondii* antibody levels and immunosenescence biomarkers in a cross-sectional sample of 584 volunteers aged 20–70 years from the Dortmund Vital Study (ClinicalTrials.gov Identifier NCT05155397) representing the regional population. One-hundred-sixty-one participants were seropositive, representing an overall 28% latent *T. gondii* seroprevalence, which did not significantly differ between males and females, but increased with age. Consequently, seropositive individuals were older than the seronegative participants. Latent *T. gondii* infection exhibited significant bivariate associations with the composite immune age index IMMAX pointing to accelerated immune aging in seropositive individuals. In addition, IMMAX increased with age and in males. However, associations of latent *T. gondii* infection with immunosenescence biomarkers disappeared when adjusting the analyses for sex and age. Moreover, the non-significant interaction between *T. gondii* status and age when predicting biomarker levels indicated that latent *T. gondii* infection did not modify the immunosenescence trend. Summarized, our results suggest that latent *T. gondii* infection is unlikely to modulate immune aging concerning cellular senescence in otherwise healthy working-age adults.

## 1. Introduction

Latent, i.e., asymptomatic toxoplasmosis caused by the infection with the parasite *Toxoplasma gondii* (*T. gondii*) is affecting large proportions of the global population with considerable variation concerning regions ranging from about 30% in Western regions to more than 50% in Southern regions [1] as well as concerning age, e.g., increasing from about 15% at 20 years to higher than 40% above 70 years in the US population [2]. Latent *T. gondii* infection might contribute to psychiatric disorders and enhance the age-related decrement in cognitive and sensory functions in older age involving pro-inflammatory cytokines [3,4]. Thus, latent *T. gondii* infection may promote the development of chronic, low-grade inflammation with aging, which is called inflammaging and supporting the pathogenesis of age-related diseases [5].

In conjunction with inflammaging, the construct ’immune age’ or ‘immunosenescence’ is used to describe the individually varying decay in functions of the immune system in the elderly [6,7,8], which may advance, not in parallel, to chronological age [9]. Inflammaging and immunosenescence are interconnected, as alterations in the aging immune system and cellular senescence can contribute to age-related inflammation [10,11,12]. While several single immune cellular senescence markers, like memory/naïve subpopulations of CD4+ and CD8+ T-cells, the CD4+/CD8+ ratio, or the frequency of CD8+ CD28− T-cells have been proposed [13,14,15,16,17,18,19,20,21], composite scores combining the information of multiple markers are considered superior in characterizing the age-related decline in immunological functions [22,23,24,25]. Here, the immune age metric IMM–AGE based on longitudinal profiles of composite multi-omics data [22] was acknowledged as one of the most advanced immunosenescence biomarkers [26,27,28,29]. In both clinical and non-clinical settings, IMM–AGE was a significant predictor of cardiovascular disease and mortality [22], the risk of sepsis in trauma patients [30], and responses to severe SARS-CoV-2 infection [31]. Recently, we established the immune age index IMMAX [32,33] approximating the comprehensive IMM–AGE metric by a few flow-cytometry-based immune cell parameters obtained from peripheral blood mononuclear cells (PBMC). IMMAX has already demonstrated its predictive capacity concerning the age-related decline in cardiorespiratory fitness [32] and work ability [34], as well as the efficacy of vaccination against SARS-CoV-2 [35] and antibody responses to SARS-CoV-2 [36].

While latent viral infections, e.g., by *Cytomegalovirus* (CMV) are known to accelerate immunological aging [37,38,39], and modulated levels of cellular senescence markers like NK cells, CD4+ and CD8+ T-cells as well as their naïve and memory sub-types have been observed under acute *T. gondii* infection [40,41,42,43,44,45], the possible role of latent *T. gondii* infection in immune aging is unknown. In addition, as immunosenescence might increase the susceptibility to infectious diseases as well as interfere with the efficacy of vaccines or pharmaceuticals [7,8,23,46], the interplay of immunological aging and *T. gondii* infection, as reported recently to be concerning ocular toxoplasmosis [47], could become relevant, e.g., for the development of vaccines or therapies against toxoplasmosis [48].

Therefore, this study aimed to examine if and how latent *T. gondii* infection will influence the levels and aging trends of immunosenescence biomarkers, especially considering the recently established composite immune age metric IMMAX [32,33] in a cross-sectional sample of about 600 participants representing the regional population by analyzing data from blood samples collected during the baseline examinations of the ongoing longitudinal Dortmund Vital Study (ClinicalTrials.gov Identifier: NCT05155397) [49].

## 2. Materials and Methods

The design of the Dortmund Vital Study including the determination of immune cell subpopulations and *T. gondii* antibodies were described in detail recently [32,49,50,51] and are briefly summarized below.

### 2.1. Dortmund Vital Study

The Dortmund Vital Study (DVS, ClinicalTrials.gov Identifier: NCT05155397) is a combined cross-sectional and longitudinal study consisting of a baseline and up to three follow-up examinations, separated by five-year intervals with a projected completion of data collection by the end of 2035. The DVS sample comprised about 600 participants, 20–70 years of age, drawn from the general healthy regional working-age population and deemed representative concerning age, genetics, and occupation, whereas females and people with a high level of education were slightly overrepresented [49]. Employing a broad definition of ‘healthy’, the DVS did not exclude individuals who were smokers, normal alcohol users (no alcohol use disorder), overweight, or persons with non-severe disease symptoms, allowing for medications with, e.g., anticoagulants, hormones, or antihypertensive and cholesterol-lowering drugs. On the other hand, people with severe neurological, cardiovascular, or oncological diseases and psychiatric disorders were excluded from participation. The comprehensive study protocol [49] included the collection of blood samples, from which *T. gondii* antibody levels and immune cell subpopulations were determined as described below in Section 2.2 and Section 2.3. This cross-sectional analysis utilized data from the baseline examinations of 584 participants with complete observations of immune cell frequencies and *T. gondii* status.

### 2.2. Immune Cell Subpopulations

**Figure 1:**
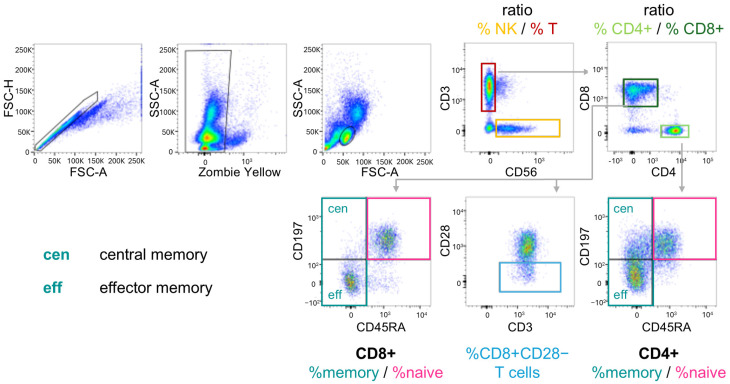
Scheme of the gating strategy for the determination of cell subpopulations by flow cytometry. PBMC were stained with the Fixable Viability Dye Zombie Yellow and for the indicated markers. NK and T-cells were separated by CD3 and CD56. T helper cells and cytotoxic T-cells were identified by CD4 and CD8 expression, respectively. Within CD8+ and CD4+ T-cells, naïve, central and effector memory cells were separated by CD45RA and CD197 staining. CD28− cells were sub-gated from CD8+ T-cells.

Peripheral venous blood was collected from DVS participants and analyzed by flow cytometry to determine a set of relative blood cell frequencies. Peripheral blood mononuclear cells (PBMC) were isolated from heparinized blood by Ficoll density gradient centrifugation (PAN-Biotech, Aidenbach, Germany) and stored at −170 °C for 1–6 months. Antibody panels were set up to analyze the lymphocytes for markers associated with aging and senescence such as NK/T-cell ratio, CD4+/CD8+ T-cell ratio, memory/naïve subpopulations of CD4+ and CD8+ T-cells (with memory cell percentages determined by adding the percentages of effector and central memory cells), and CD28− T-cells. All antibodies were individually titrated to determine the optimal dilution with the details listed in Table 1. Gating strategy is shown in Figure 1. PBMC were used immediately after thawing and were kept on ice during the staining procedure. For each panel, 0.5 × 10^6^ cells were stained with the indicated antibody cocktails for 20 min at 4 °C in the dark and then washed with FACS buffer (PBS/2% FCS). Cells were resuspended in FACS buffer and kept on ice until analysis at the same day on a BD LSRFortessa. Percentage cell frequencies were determined using the FlowJo™ v10.8 Software (BD Life Sciences). The analyses were performed according to established flow cytometry and cell sorting guidelines [52]. More specifically, cytometer setting was regularly standardized using CS&T beads including Levey-Jennings tracking for all channels. Blood samples were processed pseudonymized, i.e., without knowledge of sex, age or *T. gondii* status of the participant.

### 2.3. T. gondii Antibody Levels

Levels of the immunoglobulin G (IgG) antibody for *T. gondii* infection in the blood were determined by enzyme-linked immuno-sorbent assay (ELISA) using the IgG ELISA (IBL International, Hamburg, Germany) according to the manufacturer’s instructions. The ELISA was washed on a hydroFLEX washer and measured on a GENios plate reader system (both TECAN Group Ltd.; Maennedorf, Switzerland). The sensitivity threshold of the *T. gondii* IgG ELISA was 1.04 IU/mL. According to the manufacturer’s instructions for *T. gondii* status classification, participants with IgG-antibody levels below 30 IU/mL were assigned to the seronegative group (*T. gondii*−), whereas participants with IgG-antibody concentration above 35 IU/mL were categorized as seropositive (*T. gondii+*). One intermediate case with a serum level of 33 UI/mL was assigned to seropositive (*T. gondii+*) [50].

### 2.4. Data Analysis and Statistics

While single percentage cell frequencies (%p) were transformed to their logit(%p) = log(%p/(100% − %p)), related relative cell frequencies, e.g., memory and naïve CD8+ T-cells, exhibit a compositional structure, i.e., they inherently are negatively correlated because their sum cannot exceed 100%. This was considered by transforming such pairs to their log-ratio [53].

The composite immune age metric IMMAX combines the information of five single immunosenescence biomarkers: NK/T-cell ratio, CD4+/CD8+ T-cell ratio, the ratio of memory/naïve subpopulations in both CD4+ and CD8+ T-cells, and CD8+ CD28− T-cells. IMMAX was established as an approximation to the comprehensive marker IMM-AGE [22]. Principal component regression analysis [32] delivered predictive Equations (1) and (2), ensuring that IMMAX sores are bounded between zero and one with higher values indicating advanced immune aging. The predictors *x_i_* and coefficients *c_i_* in Equation (1) representing the contribution of the five single immunosenescence biomarkers to the composite metric IMMAX are listed in Table 2.

(1) $$logit.IMM.AGE=c_{0}+\sum_{i=1}^{5}c_{i}\times x_{i}$$

(2) IMMAX=exp(logit.IMM.AGE)/(1+exp(logit.IMM.AGE))

Trends of the immunosenescence biomarkers with age were evaluated by Pearson correlation coefficients and linear regression. The effects of *T. gondii* status (*T. gondii+* vs. *T. gondii*−) on biomarkers were assessed by Welch two-sample *t*-test and by ANCOVA adjusting for sex and age, respectively. In addition, the modifying influence of sex and *T. gondii* status on the aging trend for the separate immunosenescence biomarkers was tested by including terms for the corresponding two-way interactions of age with sex and *T. gondii* status, respectively.

## 3. Results

### 3.1. Demographics and T. gondii Seroprevalence

**Figure 2:**
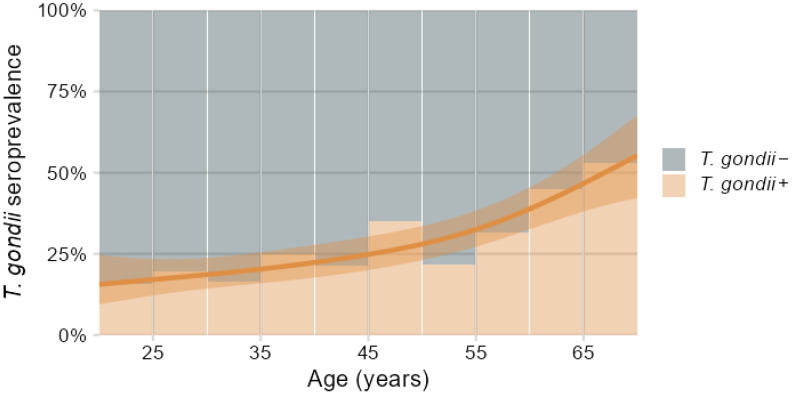
Percentage distribution of *T. gondii* seropositive (*T. gondii+*) and seronegative (*T. gondii*−) participants in relation to age binned in 5-year intervals, overlaid with spline regression predictions and 95% confidence band of *T. gondii+* prevalence depending on age fitted by generalized additive models.

One hundred and sixty-one out of 584 participants were *T. gondii* seropositive (*T. gondii+*), representing an overall 28% seroprevalence of *T. gondii* antibodies, which did not significantly differ between males and females (Table 3). There was a significant age trend with *T. gondii* seroprevalence increasing from below 20% at 20 years of age to above 50% at 70 years, as indicated in Figure 2 by the logistic regression spline function fitted by generalized additive models [54]. Consequently, the *T. gondii* seropositive group was about seven years older compared to the seronegative group (Table 3).

### 3.2. Associations of T. gondii Status with Immunosenescence Markers

**Figure 3:**
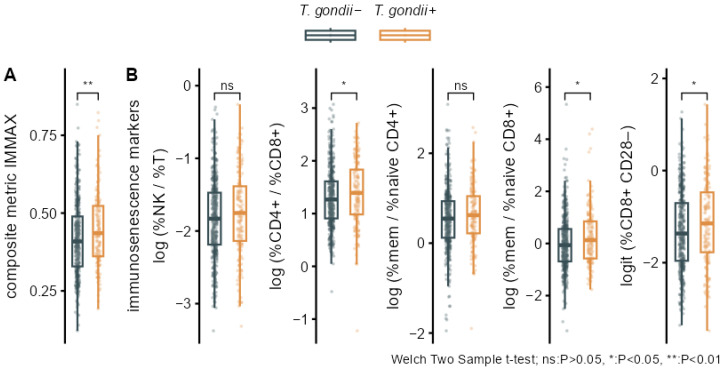
Bivariate associations of *T. gondii* status with immunosenescence biomarkers. Boxplots overlaid with individual data comparing *T. gondii+ and T. gondii*− with *p*-values from Welch two-sample *t*-tests for (**A**) the composite immune age metric IMMAX and (**B**) the single immunosenescence biomarkers.

**Figure A1:**
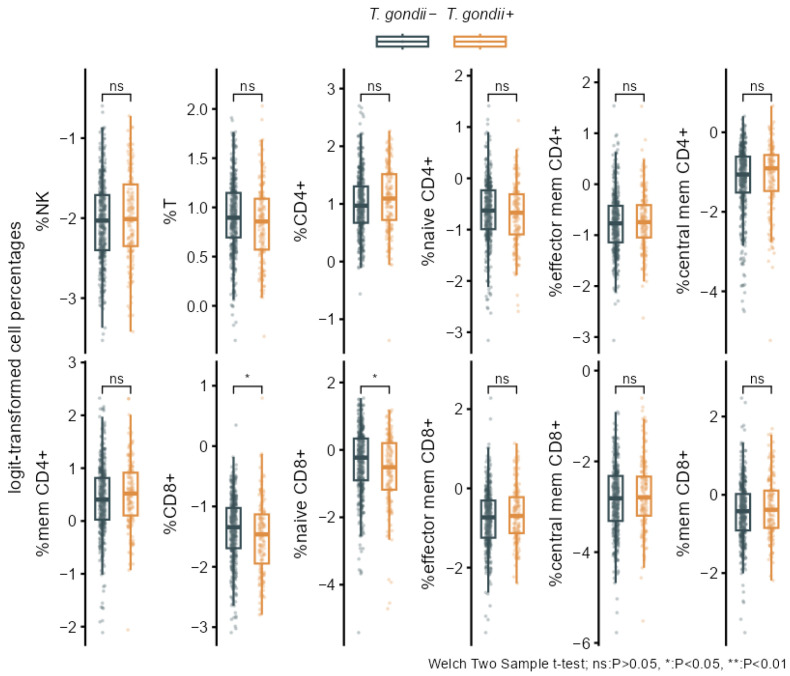
Bivariate associations of *T. gondii* status with the separate logit-transformed percentage cell frequencies. Boxplots overlaid with individual data comparing *T. gondii+ and T. gondii*− with *p*-values from Welch two-sample *t*-tests.

Bivariate analyses performed by group comparisons between seropositive (*T. gondii+*) and seronegative (*T. gondii*−) participants showed significantly increased levels of IMMAX in *T. gondii+* individuals (Figure 3A, Table 3). Higher levels in the *T. gondii+* group were also observed for the five single immunosenescence biomarkers (Figure 3B, Table 3), but these were non-significant after applying false discovery rate correction for multiple testing (Table 3), as were the corresponding group differences for cell percentages (Figure A1, Table A1).

**Figure 4:**
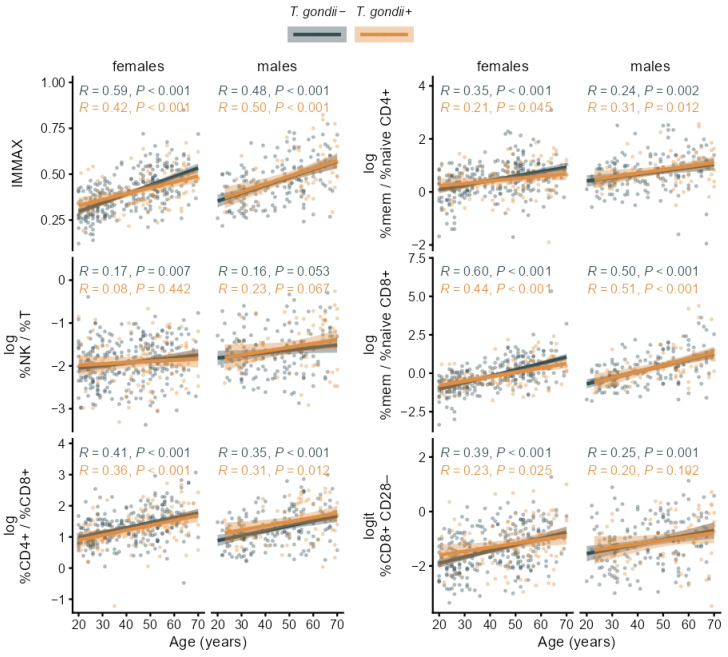
Age trends of the composite immune age metric IMMAX and single immunosenescence biomarkers in *T. gondii*+ and *T. gondii*− subgroups stratified by sex with Pearson correlation coefficients (R) and *p*-values, regression lines and 95% confidence bands.

**Figure A2:**
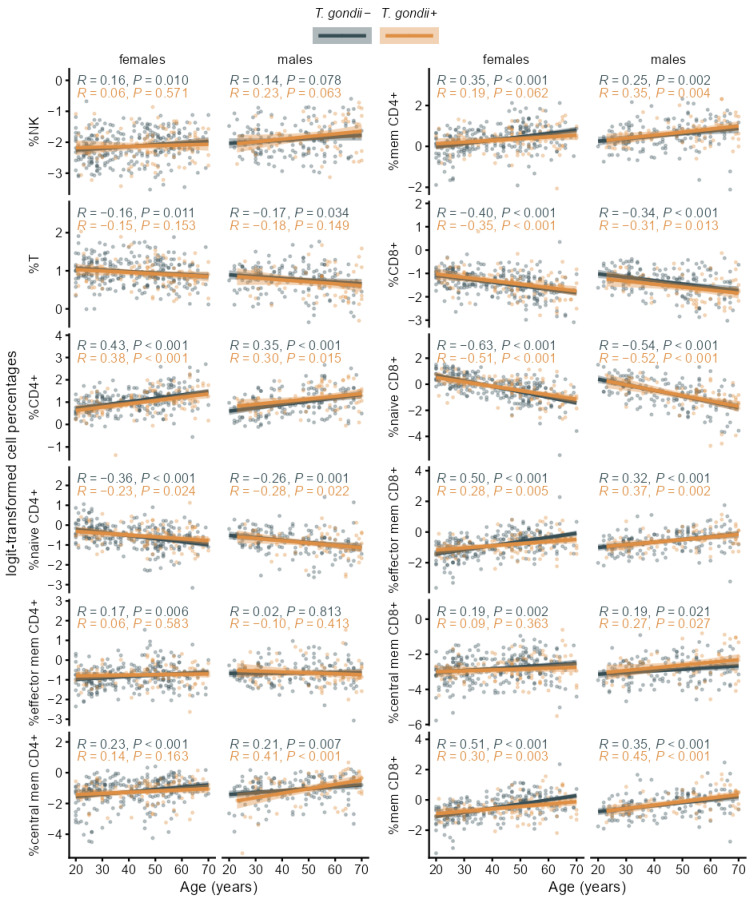
Age trends of the separate logit-transformed immune cell percentages in *T. gondii*+ and *T. gondii*− subgroups stratified by sex with Pearson correlation coefficients (R) and *p*-values, regression lines and 95% confidence bands.

As can be expected for an immune age metric, IMMAX correlated positively with age for both seropositive and seronegative participants as well in females as in males (Figure 4). This was also observed for the CD4+/CD8+ T-cell ratio and the ratio of memory/naïve subpopulations in both CD4+ and CD8+ T-cells. Similar patterns, but with lower correlation coefficients, occurred for the NK/T cell ratio and CD8+ CD28− T-cells as shown for the age trends of the single immunosenescence biomarkers in Figure 4 and the corresponding separate cell percentages in Figure A2.

**Figure 5:**
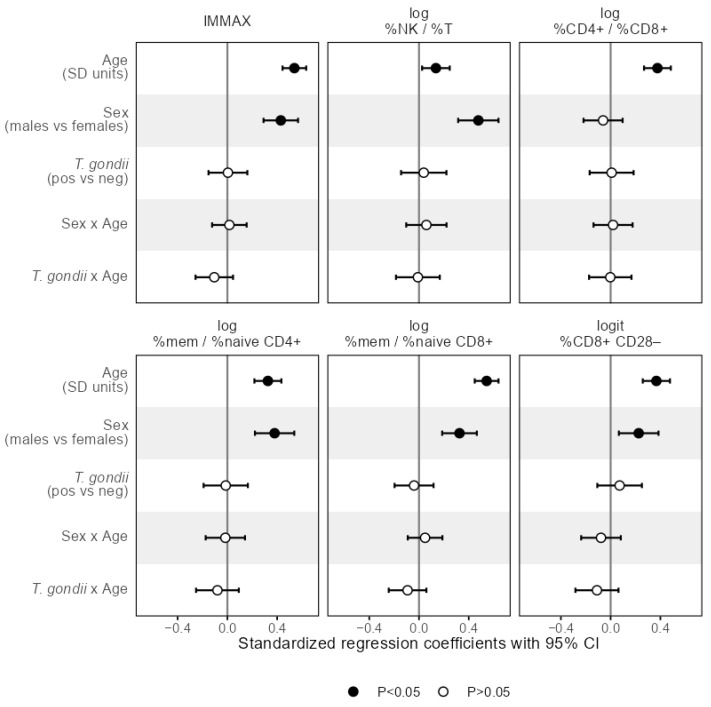
Effect sizes as standardized regression coefficients with 95% CI of the predictors age, sex, *T. gondii* status and the interaction terms denoted by the separator ‘x’ of age with sex and *T. gondii* status, respectively, from linear regression models predicting the composite immune age metric IMMAX and single immunosenescence biomarkers, respectively. Vertical reference lines indicate null effects and filled symbols mark statistically significant (*p* < 0.05) effects, respectively.

**Figure A3:**
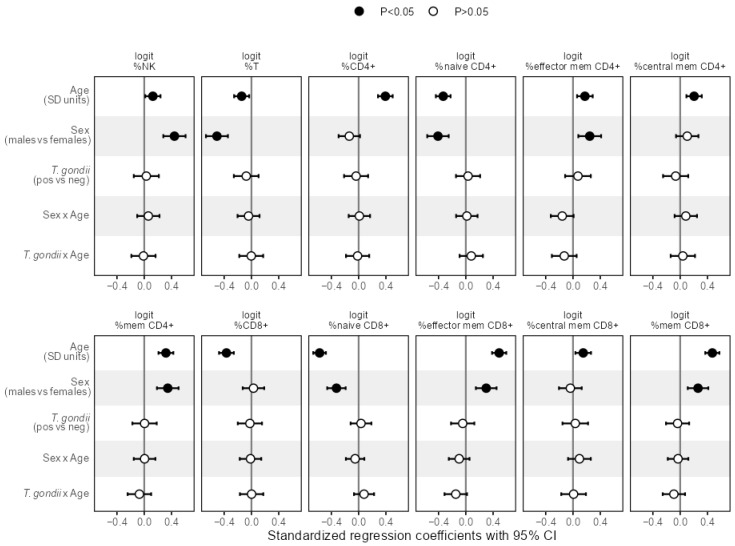
Effect sizes as standardized regression coefficients with 95% CI of the predictors age, sex, *T. gondii* status and the interactions of age with sex and *T. gondii*, respectively, from linear regression models predicting the separate logit-transformed percentage cell frequencies. Vertical reference lines indicate null effects and filled symbols mark statistically significant (*p* < 0.05) effects, respectively.

The results of the linear regression analysis in Figure 5 revealed that the slope of the age trend in IMMAX did not depend on sex, as indicated by the non-significant term for the sex-age-interaction, while IMMAX levels were increased for male participants. Analogous results were obtained for the single immunosenescence biomarkers with the CD4+/CD8+ T-cell ratio as an exception, showing a significant trend with age but no main sex effect (Figure 5), which was also observed for the single CD4+ and CD8+ cell percentages (Figure A3). Notably, central memory cell percentages for both CD4+ and CD8+ showed the same pattern with a significant age trend but no sex effect, whereas male sex added on the age effect in all remaining separate cell percentages (Figure A3).

Accounting for the influence of sex as well as for potential confounding by age being unevenly distributed between the seropositive and seronegative groups and simultaneously affecting the immunosenescence biomarkers, the bivariate group comparisons were adjusted for sex and age by ANCOVA with the results shown in Table 3 and Table A1. After adjustment, any association of *T. gondii* status with any immunosenescence biomarker and any cell percentage disappeared.

In addition, the interaction of age with *T. gondii* status was non-significant when predicting the levels of any immunosenescence biomarker and any separate cell percentage by linear regression as shown in Figure 5 and Figure A3, indicating that *T. gondii* status did not modify the age trend in IMMAX and single markers of immunosenescence.

## 4. Discussion

This study analyzed cross-sectional data concerning the association of latent *T. gondii* infection with biomarkers of immunological aging. The data were collected by taking blood samples during the baseline examinations of the ongoing longitudinal Dortmund Vital Study (DVS, ClinicalTrials.gov Identifier: NCT05155397) from 584 individuals aged 20–70 years representing the regional working-age population.

Readings from a commercial *T. gondii* specific IgG-antibody ELISA were employed to classify the participants as either seropositive (*T. gondii+*) or seronegative (*T. gondii*−) using standardized lower and upper cut-off levels of 30 IU/mL and 35 IU/mL, respectively, while an intermediate case with 33 IU/mL was considered seropositive as performed in a recent study [50]. Nevertheless, sensitivity analyses either re-classifying the intermediate case as seronegative or completely disregarding the intermediate case indicated that this did hardly influence the results presented in the Supplemental Code included as Supplementary Material. On the other hand, our primary immune age metric IMMAX combined the information from five flow-cytometry-based immunosenescence biomarkers comprising twelve single immune cell percentages, which were all reported in this study for the sake of completeness and comparison. Overall, the results for the single markers agreed with the composite metric. For example, we could confirm earlier reports on advanced immunological aging of males compared to females of same chronological age [22,32], which may be not only related to biological factors (sex differences) but also to lifestyle and environmental factors (gender differences) [55,56]. Although we observed coherent results with the different biomarkers, significant bivariate associations with *T. gondii* status only persisted for IMMAX after false discovery rate correction for multiple testing (Table 3), which might indicate a higher sensitivity of the composite metric compared to the single immunosenescence biomarkers. Therefore, we will focus on discussing the effects of latent *T. gondii* infection on the responses of IMMAX, which represents a class of comprehensive immune age metrics deeming superior to single immunosenescence biomarkers in characterizing the age-related decline in immunological functions [22,23,24,25,33].

The overall prevalence of 28% *T. gondii* seropositive individuals in the DVS sample was not influenced by sex and conformed to earlier reports concerning European and Western regions [1]. However, due to the lifelong persistence of *T. gondii* infection in humans, seroprevalence accumulated with age from below 20% at 20 years to above 50% at 70 years, as shown in Figure 2 and, similarly, previously reported for an US population [2]. This caused an uneven age distribution in the cross-sectional DVS sample with higher age for seropositive individuals requiring consideration in the subsequent evaluations.

While initial bivariate analyses concerning the association of the *T. gondii* status with IMMAX and the other immunosenescence biomarkers pointed to accelerated immune aging for seropositive individuals, significant differences between the seropositive and seronegative groups persisted only for IMMAX after false discovery rate correction for multiple testing (Table 3). Moreover, these results were susceptible and likely attributable to confounding due to the uneven age distribution in the *T. gondii+* and *T. gondii*− groups. Consequently, these associations disappeared after adjusting the analyses for sex and age by corresponding linear regression models. Additional analyses presented by the Supplemental Code included as Supplementary Material including post hoc analysis of observed power, diagnostic residual plots, and comparisons to spline regression models with non-linear age effects confirmed the adequacy of the models assuming a linear age trend presented in Table 3 and Table A1 and Figure 4, Figure 5, Figure A2 and Figure A3. This conforms to previous findings [33] showing a high agreement of the categorization into immune aging types based on IMMAX centiles estimated using either linear or non-linear age trends.

In addition, the non-significant interaction between age and *T. gondii* status when predicting IMMAX and the single immunosenescence biomarkers suggested that latent *T. gondii* infection will not modulate the observed immune aging trend. These results contrast with findings concerning the influence of latent *T. gondii* infection on sensory functions and cognition, with *T. gondii* seropositive individuals exhibiting greater impairments at old age [3,57,58,59], whereas occasionally, *T. gondii* seropositivity was beneficial in young adults, e.g., concerning hearing ability [50] and selected cognitive functions [60,61,62]. Notably, a recent study suggested that the pressure of immune surveillance on the latent form of *T. gondii* persisting in long-lived cysts might mitigate infection-induced damage and, thus, promote survival of host and parasite [63].

The significant correlation with age for IMMAX and the single markers in both *T. gondii+* and *T. gondii*− groups indicated that all markers maintained their role as an immunosenescence biomarker under latent *T. gondii* infection.

### Outlook

In contrast to our findings, recent reports on groups affected by acute sepsis [30] and severe SARS-CoV-2-infection [31] showed no age trends for immunosenescence biomarkers, which were also derived from the IMM-AGE metric [22] and, thus, bearing close resemblance to IMMAX [32]. Therefore, it will be relevant to examine whether the correlation of immunosenescence biomarkers with age observed in this study under latent *T. gondii* infection would also persist under acute or recently acquired *T. gondii* infection, which had been reported to modulate PBMC subpopulations indicative for cellular senescence, such as NK cells, CD4+ and CD8+ T-cells as well as their memory sub-types [40,41,42,43,44,45].

Such studies, which should preferably apply a longitudinal design [7,25,64], as implemented by the ongoing follow-up examinations within the DVS [49], could as well include the responses to chronic viral infections, e.g., by human *Cytomegalovirus* (CMV), which is known to advance immunosenescence [37,38,39] and might interact with *T. gondii* infection [65,66,67,68,69,70,71]. Moreover, determining and considering CMV IgG as co-variable in future analyses as illustrated by a directed acyclic graph (DAG) in the Supplemental Code included as Supplementary Material would help clarify to what extent the null effects of latent *T. gondii* infection on immune aging in our DVS baseline data might be attributable to masking due to non-observable CMV distribution differences in the *T. gondii* seropositive and seronegative subgroups, respectively. This could be supplemented by considering inflammation markers like C-reactive protein (CRP) or cytokines, where a recent analysis of a different database suggested that *T. gondii* status would not modify the age-related trend of selected pro-inflammatory cytokines like interleukin-6 (IL-6) or tumor necrosis factor TNF-α [60].

## 5. Conclusions

The results of this study underpin the necessity for adjusting bivariate associations in cross-sectional data for confounding and effect-modifying factors, reinforcing the postulated urgent need for data from longitudinal studies concerning immune senescence. The ongoing follow-up examinations within the DVS will facilitate studying, longitudinally, how immunosenescence markers will respond to incident *T. gondii* infection acquired during the 5-year follow-up period, where the availability of corresponding data including information on CMV and cytokines is anticipated by the end of 2026.

At present, the outcomes of this cross-sectional analysis suggest that the observed immunosenescence probably reflects an age-related physiological process and the latent stage of *T. gondii* will be unlikely to modulate immune aging in terms of cellular senescence in otherwise healthy working-age adults. If confirmed by prospective longitudinal studies, this trait of the latent form of *T. gondii* would, if not promote, at least not compromise survival of host and parasite.

## Figures and Tables

**Table 1:** Materials for PBMC staining, including antigens, antibody clones and coupled fluorochromes, distributors and antibody dilution used to stain 0.5 × 10^6^ PBMC.

Antigen	Clone	Fluorochrome	Company	Dilution 1/x
CD3	UCHT1	BV510	BD Horizon™ (Franklin Lakes, NJ, USA)	400
live/dead		zombie Yellow	Biolegend (San Diego, CA, USA)	1000
CD8	RPA-T8	FITC	BD Pharmingen™ (Franklin Lakes, NJ, USA)	200
CD28	CD28.2	PerCP-Cy™ 5.5	BD Pharmingen™	100
CD57	NK-1	PE	BD Pharmingen™	800
CD56	B159	PE-CF594	BD Pharmingen™	100
CD197 (CCR7)	150503	Alexa Fluor^®^ 647	BD Pharmingen™	50
CD4	RPA-T4	APC-H7	BD Pharmingen™	100
CD45RA	HI100	Alexa Fluor^®^ 700	BD Pharmingen™	400

**Table 2:** Predictors (x_i_) and coefficients (c_i_) in Equation (1) for calculating the composite IMMAX metric from single immunosenescence biomarkers.

Index i	Predictor *x_i_*	Coefficient *c_i_*
0	1 (constant)	0.024798
1	log (%NK/%T)	0.154033
2	log (%mem/%naive CD4+)	0.236631
3	log (%CD4+/%CD8+)	0.063959
4	log (%mem/%naive CD8+)	0.235855
5	logit (%CD8+ CD28−)	0.227827

Abbreviations: CD4+: CD4 positive T-cell, CD8+ CD28−: CD28 negative CD8 positive T-cell, CD8+: CD8 positive T-cell, NK: Natural Killer cell, T: T-cell, logit: logit of percentage p% as logit(p%) = log(p%)/log(100% − p%), mem: memory T-cell, naive: naive T-cell.

**Table 3:** Demographics and immunosenescence biomarkers stratified by *T. gondii* status.

Characteristics	*T. gondii*− *N* = 423 (72%) ^1^	*T. gondii*+ *N* = 161 (28%) ^1^	P ^2^	q-Value ^2^	∆_adj_ (95% CI) ^3^	P_adj_ ^3^
**Demographics**						
Sex			0.406	0.406		
female	268 (74%)	96 (26%)				
male	155 (70%)	65 (30%)				
Age (years)	42 (14)	49 (14)	**<0.001**	**<0.001**		
**Immunosenescence biomarkers**						
IMMAX	0.42 (0.12)	0.45 (0.12)	**0.007**	**0.029**	−0.02 (−0.17, 0.14)	0.823
log (%NK/%T)	−1.83 (0.53)	−1.76 (0.58)	0.173	0.197	0.04 (−0.14, 0.22)	0.676
log (%CD4+/%CD8+)	1.29 (0.56)	1.41 (0.62)	**0.048**	0.077	0.01 (−0.16, 0.18)	0.928
log (%mem/%naive CD4+)	0.54 (0.68)	0.63 (0.65)	0.159	0.197	−0.03 (−0.20, 0.14)	0.730
log (%mem/%naive CD8+)	0.00 (1.01)	0.23 (1.06)	**0.023**	0.061	−0.06 (−0.21, 0.10)	0.460
logit (%CD8+ CD28−)	−1.32 (0.85)	−1.14 (0.92)	**0.034**	0.067	0.05 (−0.13, 0.22)	0.600

^1^ Frequency (%) or mean (SD) ^2^ *p*-values from Pearson’s Chi-squared test or Welch two-sample *t*-test with q-values from false discovery rate correction for multiple testing (significant values in bold) ^3^ ∆_adj_: *T. gondii*+ effect as standardized mean differences adjusted for sex and age by ANCOVA with 95% CI and *p*-value. Abbreviations as in Table 2 plus CI: Confidence Interval, IMMAX: composite metric immune age index, *T. gondii*+: *T. gondii* seropositive, *T. gondii*−: *T. gondii* seronegative.

## Data Availability

The original data presented in the study are openly available in a repository at OSF at https://osf.io/9vg2z, (accessed on 17 December 2025) while supplemental code reproducing the core results and additional analyses is available at https://osf.io/xzw6p (accessed on 17 December 2025). In addition, the DVS data presented in this study are available on request from the corresponding author due to the research data policy of the DVS as outlined in the Research Data Management section of the study protocol [49].

## Supplementary Materials

The following supporting information can be downloaded at: https://www.mdpi.com/article/10.3390/biom16010055/s1, Supplemental Code for reproducing the core results from the provided study data and additional analyses with output generated using R version 4.5.1 [72].

## Abbreviations

The following abbreviations are used in this manuscript:

ANCOVA	Analysis of co-variance
CD4+	CD4 positive T-cells
CD8+	CD8 positive T-cells
CD8+ CD28−	CD28 negative CD8 positive T-cells
CMV	*Cytomegalovirus*
CI	Confidence Interval
DVS	Dortmund Vital Study
ELISA	Enzyme-Linked Immuno-Sorbent Assay
IMMAX	Composite metric IMMune Age indeX
IgG	Immunoglobulin G antibody
NK	Natural Killer cells
PBMC	Peripheral Blood Mononuclear Cells
q	*p*-value corrected for false discovery rate in multiple testing
SD	Standard Deviation
T	T-cells
*T. gondii*	*Toxoplasma gondii*
*T. gondii*+	*T. gondii* seropositive
*T. gondii*−	*T. gondii* seronegative
%cen mem	Percentage of central memory T-cells
%eff mem	Percentage of effector memory T-cells
%mem	Percentage of memory T-cells
%naive	Percentage of naïve T-cells

## Appendix A

This appendix contains supplemental analyses concerning the association of *T. gondii* status with the single immune cell percentages presented as Table A1, Figure A1, Figure A2 and Figure A3.

**Table A1:** Means (SD) of logit-transformed immune cell percentages stratified by *T. gondii* status with group comparisons performed by Welch *t*-test and by ANCOVA adjusting for sex and age, respectively.

Logit-Transformed Cell Percentages	*T. gondii*− *N* = 423 ^1^	*T. gondii*+ *N* = 161 ^1^	P ^2^	q-Value ^2^	∆_adj_ (95% CI) ^3^	P_adj_^3^
%NK	−2.05 (0.51)	−1.99 (0.55)	0.226	0.271	0.03 (−0.15, 0.21)	0.750
%T	0.90 (0.37)	0.84 (0.39)	0.065	0.243	−0.08 (−0.26, 0.10)	0.386
%CD4+	1.01 (0.52)	1.09 (0.56)	0.130	0.251	−0.04 (−0.21, 0.13)	0.648
%naive CD4+	−0.64 (0.62)	−0.71 (0.60)	0.219	0.271	0.05 (−0.12, 0.22)	0.571
%eff mem CD4+	−0.76 (0.59)	−0.71 (0.57)	0.340	0.371	0.04 (−0.14, 0.23)	0.661
%cen mem CD4+	−1.19 (0.85)	−1.13 (0.87)	0.465	0.465	−0.06 (−0.24, 0.12)	0.543
%mem CD4+	0.41 (0.67)	0.50 (0.64)	0.104	0.250	−0.01 (−0.18, 0.16)	0.907
%CD8+	−1.38 (0.54)	−1.50 (0.60)	**0.037**	0.222	−0.02 (−0.20, 0.15)	0.798
%naive CD8+	−0.37 (1.00)	−0.62 (1.05)	**0.012**	0.141	0.05 (−0.10, 0.20)	0.509
%eff mem CD8+	−0.76 (0.74)	−0.66 (0.69)	0.146	0.251	−0.08 (−0.25, 0.09)	0.351
%cen mem CD8+	−2.85 (0.73)	−2.75 (0.78)	0.177	0.266	0.04 (−0.15, 0.22)	0.685
%mem CD8+	−0.43 (0.76)	−0.31 (0.76)	0.081	0.243	−0.06 (−0.22, 0.11)	0.489

^1^ Mean (SD) ^2^ *p*-values from Welch two-sample *t*-test with q-values from false discovery rate correction for multiple testing (significant values in bold) ^3^ ∆_adj_: *T. gondii*+ effect as standardized mean differences adjusted for sex and age by ANCOVA with 95% CI and *p*-value. Abbreviations: CD4+: CD4 positive T-cell, CD8+: CD8 positive T-cell, CI: Confidence Interval, NK: Natural Killer cell, *T. gondii+*: *T. gondii* seropositive, *T. gondii*−: *T. gondii* seronegative, T: T-cell, logit: logit of percentage p% as logit(p%) = log(p%)/log(100% − p%), mem: memory T-cell, cen mem: central memory T-cell, eff mem: effector memory T-cell, naive: naive T-cell.

## References

[1] Robert-Gangneux F., Dardé M.-L. (2012). Epidemiology of and Diagnostic Strategies for Toxoplasmosis. Clin. Microbiol. Rev..

[2] Jones J.L., Kruszon-Moran D., Wilson M., McQuillan G., Navin T., McAuley J.B. (2001). *Toxoplasma gondii* Infection in the United States: Seroprevalence and Risk Factors. Am. J. Epidemiol..

[3] Colzato L., Zhang W., Beste C., Stock A.-K. (2021). Dissociating direct and indirect effects: A theoretical framework of how latent toxoplasmosis affects cognitive profile across the lifespan. Neurobiol. Aging.

[4] Naranjo-Galvis C.A., Cardona-Londoño K.Y., Orrego-Cardozo M., Elcoroaristizabal-Martín X. (2022). *Toxoplasma gondii* infection and peripheral-blood gene expression profiling of older people reveals dysregulation of cytokines and identifies hub genes as potential therapeutic targets. Heliyon.

[5] Franceschi C., Garagnani P., Parini P., Giuliani C., Santoro A. (2018). Inflammaging: A new immune–metabolic viewpoint for age-related diseases. Nat. Rev. Endocrinol..

[6] Pawelec G. (2018). Age and immunity: What is “immunosenescence”?. Exp. Gerontol..

[7] Pawelec G., Bronikowski A., Cunnane S.C., Ferrucci L., Franceschi C., Fülöp T., Gaudreau P., Gladyshev V.N., Gonos E.S., Gorbunova V. (2020). The conundrum of human immune system “senescence”. Mech. Ageing Dev..

[8] Fu Y., Wang B., Alu A., Hong W., Lei H., He X., Shi H., Cheng P., Yang X. (2025). Immunosenescence: Signaling pathways, diseases and therapeutic targets. Signal Transduct. Target. Ther..

[9] Xu W., Wong G., Hwang Y.Y., Larbi A. (2020). The untwining of immunosenescence and aging. Semin. Immunopathol..

[10] Teissier T., Boulanger E., Cox L.S. (2022). Interconnections between Inflammageing and Immunosenescence during Ageing. Cells.

[11] Aw D., Silva A.B., Palmer D.B. (2007). Immunosenescence: Emerging challenges for an ageing population. Immunology.

[12] Fard M.T., Savage K.M., Stough C.K. (2022). Peripheral inflammation marker relationships to cognition in healthy older adults—A systematic review. Psychoneuroendocrinology.

[13] Appay V., Sauce D. (2014). Naive T cells: The crux of cellular immune aging?. Exp. Gerontol..

[14] Garrido-Rodríguez V., Herrero-Fernández I., Castro M.J., Castillo A., Rosado-Sánchez I., Galvá M.I., Ramos R., Olivas-Martínez I., Bulnes-Ramos Á., Cañizares J. (2021). Immunological features beyond CD4/CD8 ratio values in older individuals. Aging.

[15] Ligotti M.E., Aiello A., Accardi G., Aprile S., Bonura F., Bulati M., Gervasi F., Giammanco G.M., Pojero F., Zareian N. (2021). Analysis of T and NK cell subsets in the Sicilian population from young to supercentenarian: The role of age and gender. Clin. Exp. Immunol..

[16] Ramasubramanian R., Meier H.C.S., Vivek S., Klopack E., Crimmins E.M., Faul J., Nikolich-Žugich J., Thyagarajan B. (2022). Evaluation of T-cell aging-related immune phenotypes in the context of biological aging and multimorbidity in the Health and Retirement Study. Immun. Ageing.

[17] Rodriguez I.J., Lalinde Ruiz N., Llano León M., Martínez Enríquez L., Montilla Velásquez M.d.P., Ortiz Aguirre J.P., Rodríguez Bohórquez O.M., Velandia Vargas E.A., Hernández E.D., Parra López C.A. (2021). Immunosenescence Study of T Cells: A Systematic Review. Front. Immunol..

[18] Brzezińska A., Magalska A., Szybińska A., Sikora E. (2004). Proliferation and apoptosis of human CD8+CD28+ and CD8+CD28− lymphocytes during aging. Exp. Gerontol..

[19] Fagnoni F.F., Vescovini R., Passeri G., Bologna G., Pedrazzoni M., Lavagetto G., Casti A., Franceschi C., Passeri M., Sansoni P. (2000). Shortage of circulating naive CD8+ T cells provides new insights on immunodeficiency in aging. Blood.

[20] Vescovini R., Fagnoni F.F., Telera A.R., Bucci L., Pedrazzoni M., Magalini F., Stella A., Pasin F., Medici M.C., Calderaro A. (2014). Naïve and memory CD8 T cell pool homeostasis in advanced aging: Impact of age and of antigen-specific responses to *Cytomegalovirus*. AGE.

[21] Huff W.X., Kwon J.H., Henriquez M., Fetcko K., Dey M. (2019). The Evolving Role of CD8+CD28− Immunosenescent T Cells in Cancer Immunology. Int. J. Mol. Sci..

[22] Alpert A., Pickman Y., Leipold M., Rosenberg-Hasson Y., Ji X., Gaujoux R., Rabani H., Starosvetsky E., Kveler K., Schaffert S. (2019). A clinically meaningful metric of immune age derived from high-dimensional longitudinal monitoring. Nat. Med..

[23] Li W., Zhang Z., Kumar S., Botey-Bataller J., Zoodsma M., Ehsani A., Zhan Q., Alaswad A., Zhou L., Grondman I. (2025). Single-cell immune aging clocks reveal inter-individual heterogeneity during infection and vaccination. Nat. Aging.

[24] Rizzo L.B., Swardfager W., Maurya P.K., Graiff M.Z., Pedrini M., Asevedo E., Cassinelli A.C., Bauer M.E., Cordeiro Q., Scott J. (2018). An immunological age index in bipolar disorder: A confirmatory factor analysis of putative immunosenescence markers and associations with clinical characteristics. Int. J. Methods Psychiatr. Res..

[25] Rutledge J., Oh H., Wyss-Coray T. (2022). Measuring biological age using omics data. Nat. Rev. Genet..

[26] Li S., Wang K., Wu J., Zhu Y. (2025). The immunosenescence clock: A new method for evaluating biological age and predicting mortality risk. Ageing Res. Rev..

[27] Henrickson S.E. (2019). Is your immune system over the hill?. Sci. Immunol..

[28] Dolan M., Libby K.A., Ringel A.E., van Galen P., McAllister S.S. (2025). Ageing, immune fitness and cancer. Nat. Rev. Cancer.

[29] Franceschi C., Olivieri F., Moskalev A., Ivanchenko M., Santoro A. (2025). Toward precision interventions and metrics of inflammaging. Nat. Aging.

[30] Foster M.A., Bentley C., Hazeldine J., Acharjee A., Nahman O., Shen-Orr S.S., Lord J.M., Duggal N.A. (2022). Investigating the potential of a prematurely aged immune phenotype in severely injured patients as predictor of risk of sepsis. Immun. Ageing.

[31] Lord J.M., Veenith T., Sullivan J., Sharma-Oates A., Richter A.G., Greening N.J., McAuley H.J.C., Evans R.A., Moss P., Moore S.C. (2024). Accelerated immune ageing is associated with COVID-19 disease severity. Immun. Ageing.

[32] Bröde P., Claus M., Gajewski P.D., Getzmann S., Golka K., Hengstler J.G., Wascher E., Watzl C. (2022). Calibrating a Comprehensive Immune Age Metric to Analyze the Cross Sectional Age-Related Decline in Cardiorespiratory Fitness. Biology.

[33] Bröde P., Claus M., Gajewski P.D., Getzmann S., Wascher E., Watzl C. (2023). From Immunosenescence to Aging Types–Establishing Reference Intervals for Immune Age Biomarkers by Centile Estimation. Int. J. Mol. Sci..

[34] Gajewski P.D., Rieker J.A., Athanassiou G., Bröde P., Claus M., Golka K., Hengstler J.G., Kleinsorge T., Nitsche M.A., Reinders J. (2023). A Systematic Analysis of Biological, Sociodemographic, Psychosocial, and Lifestyle Factors Contributing to Work Ability Across the Working Life Span: Cross-sectional Study. JMIR Form. Res..

[35] Claus M., Bröde P., Urlaub D., Wolfsdorff N., Watzl C. (2022). Investigation of the relationship between Immune Age and Vaccination against SARS-CoV-2. Eur. J. Immunol..

[36] Davies M., Denise H., Day M., Henson S.M., Scotton C.J., Harries L.W. (2025). Immune age is correlated with decreased TCR clonal diversity and antibody response to SARS-CoV-2. Sci. Rep..

[37] Yan Z., Maecker H.T., Brodin P., Nygaard U.C., Lyu S.C., Davis M.M., Nadeau K.C., Andorf S. (2021). Aging and CMV discordance are associated with increased immune diversity between monozygotic twins. Immun. Ageing.

[38] Pawelec G. (2022). Latent CMV makes older adults less naive. eBioMedicine.

[39] Pawelec G., Derhovanessian E. (2011). Role of CMV in immune senescence. Virus Res..

[40] Khan I.A., Moretto M. (2022). Immune responses to *Toxoplasma gondii*. Curr. Opin. Immunol..

[41] Khan I.A., Ouellette C., Chen K., Moretto M. (2019). Toxoplasma: Immunity and Pathogenesis. Curr. Clin. Microbiol. Rep..

[42] Moretto M.M., Chen J., Meador M., Phan J., Khan I.A. (2023). A Lower Dose of Infection Generates a Better Long-Term Immune Response against *Toxoplasma gondii*. ImmunoHorizons.

[43] Gazzinelli R., Xu Y., Hieny S., Cheever A., Sher A. (1992). Simultaneous depletion of CD4+ and CD8+ T lymphocytes is required to reactivate chronic infection with *Toxoplasma gondii*. J. Immunol..

[44] Chu H.H., Chan S.-W., Gosling J.P., Blanchard N., Tsitsiklis A., Lythe G., Shastri N., Molina-París C., Robey E.A. (2016). Continuous Effector CD8+ T Cell Production in a Controlled Persistent Infection Is Sustained by a Proliferative Intermediate Population. Immunity.

[45] Khan I.A., Hwang S., Moretto M. (2019). *Toxoplasma gondii*: CD8 T Cells Cry for CD4 Help. Front. Cell. Infect. Microbiol..

[46] Crooke S.N., Ovsyannikova I.G., Poland G.A., Kennedy R.B. (2019). Immunosenescence and human vaccine immune responses. Immun. Ageing.

[47] Eraghi A.T., Garweg J.G., Pleyer U. (2024). The role of age in ocular toxoplasmosis: Clinical signs of immunosenescence and inflammaging. Front. Med..

[48] Qiu Y., Wang W., Wang Q., Xu J., Dai G., Bai Y., Zhang J. (2025). Activity Evaluation and Mode of Action of ICA Against *Toxoplasma gondii* In Vitro. Biomolecules.

[49] Gajewski P.D., Getzmann S., Bröde P., Burke M., Cadenas C., Capellino S., Claus M., Genç E., Golka K., Hengstler J.G. (2022). Impact of Biological and Lifestyle Factors on Cognitive Aging and Work Ability in the Dortmund Vital Study: Protocol of an Interdisciplinary, Cross-sectional, and Longitudinal Study. JMIR Res. Protoc..

[50] Getzmann S., Golka K., Bröde P., Reinders J., Kadhum T., Hengstler J.G., Wascher E., Gajewski P.D. (2024). Chronic *Toxoplasma gondii* Infection Modulates Hearing Ability across the Adult Life Span. Life.

[51] Claus M., Dychus N., Ebel M., Damaschke J., Maydych V., Wolf O.T., Kleinsorge T., Watzl C. (2016). Measuring the immune system: A comprehensive approach for the analysis of immune functions in humans. Arch. Toxicol..

[52] Cossarizza A., Chang H.-D., Radbruch A., Abrignani S., Addo R., Akdis M., Andrä I., Andreata F., Annunziato F., Arranz E. (2021). Guidelines for the use of flow cytometry and cell sorting in immunological studies (third edition). Eur. J. Immunol..

[53] van den Boogaart K.G., Tolosana-Delgado R. (2013). Analyzing Compositional Data with R.

[54] Wood S.N. (2017). Generalized Additive Models: An Introduction with R.

[55] Calabrò A., Accardi G., Aiello A., Caruso C., Candore G. (2023). Sex and gender affect immune aging. Front. Aging.

[56] Hirokawa K., Utsuyama M., Hayashi Y., Kitagawa M., Makinodan T., Fulop T. (2013). Slower immune system aging in women versus men in the Japanese population. Immun. Ageing.

[57] Gajewski P.D., Falkenstein M., Hengstler J.G., Golka K. (2014). *Toxoplasma gondii* impairs memory in infected seniors. Brain Behav. Immun..

[58] Mendy A., Vieira E.R., Albatineh A.N., Gasana J. (2015). Immediate rather than delayed memory impairment in older adults with latent toxoplasmosis. Brain Behav. Immun..

[59] Song G., Zhao Q., Chen H., Li M., Zhang Z., Qu Z., Yang C., Lin X., Ma W., Standlee C.R. (2024). *Toxoplasma gondii* seropositivity and cognitive functioning in older adults: An analysis of cross-sectional data of the National Health and Nutrition Examination Survey 2011–2014. BMJ Open.

[60] Gajewski P.D., Bröde P., Claus M., Golka K., Hengstler J.G., Reinders J., Watzl C., Wascher E., Getzmann S. (2025). Changes of cognitive functions and proinflammatory cytokines across the lifespan in latent *Toxoplasma gondii* infection. Brain Behav. Immun. Health.

[61] Stock A.-K., Dajkic D., Köhling H.L., von Heinegg E.H., Fiedler M., Beste C. (2017). Humans with latent toxoplasmosis display altered reward modulation of cognitive control. Sci. Rep..

[62] Stock A.-K., Heintschel von Heinegg E., Köhling H.-L., Beste C. (2014). Latent *Toxoplasma gondii* infection leads to improved action control. Brain Behav. Immun..

[63] Eberhard J.N., Shallberg L.A., Winn A., Chandrasekaran S., Giuliano C.J., Merritt E.F., Willis E., Konradt C., Christian D.A., Aldridge D.L. (2025). Immune targeting and host-protective effects of the latent stage of *Toxoplasma gondii*. Nat. Microbiol..

[64] Ahadi S., Zhou W., Schüssler-Fiorenza Rose S.M., Sailani M.R., Contrepois K., Avina M., Ashland M., Brunet A., Snyder M. (2020). Personal aging markers and ageotypes revealed by deep longitudinal profiling. Nat. Med..

[65] Andreou D., Steen N.E., Jørgensen K.N., Ueland T., Wortinger L.A., Mørch-Johnsen L., Drabløs I., Calkova T., Yolken R.H., Andreassen O.A. (2024). Increased Herpes simplex virus 1, *Toxoplasma gondii* and *Cytomegalovirus* antibody concentrations in severe mental illness. Transl. Psychiatry.

[66] Fulop T., Larbi A., Pawelec G. (2013). Human T Cell Aging and the Impact of Persistent Viral Infections. Front. Immunol..

[67] Gelderman A.H., Grimley P.M., Lunde M.N., Rabson A.S. (1968). *Toxoplasma gondii* and *Cytomegalovirus*: Mixed Infection by a Parasite and a Virus. Science.

[68] Pomeroy C., Kline S., Jordan M.C., Filice G.A. (1989). Reactivation of *Toxoplasma gondii* by *Cytomegalovirus* Disease in Mice: Antimicrobial Activities of Macrophages. J. Infect. Dis..

[69] Ross D.S., Jones J.L., Lynch M.F. (2006). Toxoplasmosis, *Cytomegalovirus*, Listeriosis, and Preconception Care. Matern. Child. Health J..

[70] Frye M.A., Coombes B.J., McElroy S.L., Jones-Brando L., Bond D.J., Veldic M., Romo-Nava F., Bobo W.V., Singh B., Colby C. (2019). Association of *Cytomegalovirus* and *Toxoplasma gondii* Antibody Titers with Bipolar Disorder. JAMA Psychiatry.

[71] Gratama J.W., Fridell E., Lenkei R., Oosterveer M.A.P., Ljungström I., Tanke H.J., Linde A. (1989). Correlation between *Cytomegalovirus* and *Toxoplasma gondii* Serology and Lymphocyte Phenotypes in Peripheral Blood and Cord Blood. Scand. J. Infect. Dis..

[72] R Core Team (2025). R: A Language and Environment for Statistical Computing.

