# Supplementary material for: Latent Toxoplasma gondii Infection Does Not Modulate Immune Aging in a Cross-Sectional Working-Age Population Study
Source: Biomolecules. 2025 Dec 30;16(1):55. doi: 10.3390/biom16010055 (PMC12838790; doi:10.3390/biom16010055)
Supplement: Supplementary file 1 [file biomolecules-16-00055-s001.zip › biomolecules-3972705-supplementary.pdf]

Supplemental R code to

Latent *Toxoplasma gondii* infection does not modulate immune aging in a cross-sectional working-age population study

Peter Bröde[1], Maren Claus[1], Stephan Getzmann[1], Klaus Golka[1], Jan G. Hengstler[1], Jörg Reinders[1], Edmund Wascher[1,2], Carsten Watzl[1] and Patrick D. Gajewski[1,*]

[1] Leibniz Research Centre for Working Environment and Human Factors at TU Dortmund (IfADo), Ardeystraße 67, D-44139 Dortmund, Germany

[2] German Center for Mental Health (DZPG), partner site Bochum/Marburg

* Correspondence: gajewski@ifado.de; Tel.: +49 231 1084 383

Submitted to Biomolecules, 2025-12-17

Available at https://osf.io/xzw6p

```
library(tidyverse)
library(gtsummary)
library(cowplot)
library(ggstats)
library(pwrss)
library(splines)
library(gridGraphics)
library(mgcv)
library(tidygam)
library(ggdag)

packages=c("cowplot","ggdag","ggstats","gridGraphics","gtsummary","mgcv",
           "pwrss","report","splines","tidygam","tidyverse")
# read data from OSF repository
immune.age.markers<-c(
  "log.NK.T",
  "log.T.CD4pos.CD8pos",
  "log.T.CD4.mem.naive",
  "log.T.CD8.mem.naive",
  "logit.CD8posCD28neg.T.cells"
)
#
my_url <- "https://osf.io/9vg2z/download"
DVS.dat=read.csv(my_url) %>%
  mutate(
    sex=factor(ifelse(Female==1,"female","male"))
  ) %>%
  na.omit() %>%
  select(T.gondii.IgG,sex,age,IMMAX,all_of(immune.age.markers))
```

```r
# Table 1
# set compact theme
set_gtsummary_theme(theme_gtsummary_compact())

# Bivariate analyses (left part of Table 1)
DVS.dat %>%
  mutate(
    T.gondii=factor(ifelse(T.gondii.IgG==0.5,1,T.gondii.IgG),
                    levels = c(0,1),
                    labels = c("neg","pos"))
  ) %>%
  na.omit() %>%
  select(T.gondii,sex,age,IMMAX,all_of(immune.age.markers)) %>%
  tbl_summary(
    by=T.gondii,
    statistic = list(all_continuous() ~ "{mean} ({sd})"),
    percent = "row",
    missing_text = "#missing"
  )|>
  add_p(test=list(all_continuous() ~ "t.test"),
        pvalue_fun = ~style_pvalue(., digits = 3)) |>
  bold_p(q=FALSE) %>%
  add_q()%>%
  bold_p(q=TRUE)  |>
  add_variable_group_header(
    header = "Demographics",
    variables = c(sex,age)
  ) |>
  add_variable_group_header(
    header = "Immunosenescence biomarkers",
    variables = c(all_of(immune.age.markers),IMMAX)
  ) |>
  modify_footnote_header("Frequency (%) or Mean (SD)", columns = all_stat_cols()) |>
  modify_header(label ~ "**Characteristics**",p.value = "**P**",
                all_stat_cols() ~ "**{level}**\nN = {n} ({style_percent(p)}%)") %>%
  as_flex_table()
```

**Characteristics**	**neg**<br>N = 423 (72%)[1]	**pos**<br>N = 161 (28%)[1]	**P**[2]	**q-value**[3]
Demographics				
sex			0.406	0.406
female	268 (74%)	96 (26%)		
male	155 (70%)	65 (30%)		
age	42 (14)	49 (14)	**<0.001**	**<0.001**
Immunosenescence biomarkers				
IMMAX	0.42 (0.12)	0.45 (0.12)	**0.007**	**0.029**
log.NK.T	-1.83 (0.53)	-1.76 (0.58)	0.173	0.197
log.T.CD4pos.CD8pos	1.29 (0.56)	1.41 (0.62)	**0.048**	0.077
log.T.CD4.mem.naive	0.54 (0.68)	0.63 (0.65)	0.159	0.197
log.T.CD8.mem.naive	0.00 (1.01)	0.23 (1.06)	**0.023**	0.061
logit.CD8posCD28neg.T.cells	-1.32 (0.85)	-1.14 (0.92)	**0.034**	0.067

[1]Frequency (%) or Mean (SD)
[2]Pearson's Chi-squared test; Welch Two Sample t-test
[3]False discovery rate correction for multiple testing

```r
# ANCOVA adjusting for sex and age (right part of Table 1)
my_Delta_adj <- function(data, variable, by, ...) {
  lm(scale(data[[variable]]) ~  data[[by]] + scale(data[["age"]])+ data[["sex"]]) |>
    broom::tidy(conf.int=TRUE) %>%
    filter(substr(term,1,10)=="data[[by]]") %>%
    dplyr::mutate(
      p.adj={style_pvalue(p.value,digits=3)},
      Delta_95CI_adj = glue::glue("{style_sigfig(estimate)}
({style_sigfig(conf.low)}, {style_sigfig(conf.high)})")
    ) %>%
    select(Delta_95CI_adj, p.value)
}
#
DVS.dat %>%
  mutate(
    T.gondii=factor(ifelse(T.gondii.IgG==0.5,1,T.gondii.IgG),
                    levels = c(0,1),
                    labels = c("neg","pos"))
  ) %>%
  na.omit() %>%
  select(T.gondii,sex,age,IMMAX,all_of(immune.age.markers)) %>%
  tbl_summary(
    by=T.gondii,
    include = -c(sex,age),
    statistic = list(
      all_continuous() ~ "{mean} ({sd})"
    ),
    percent = "row",
    missing_text = "#missing"
  )|>
  add_stat(fns = everything() ~ my_Delta_adj) |>
  modify_fmt_fun(p.value = label_style_pvalue(digits = 3))%>%
  bold_p(q=FALSE) %>%
  add_variable_group_header(
    header = "Immunosenescence biomarkers",
    variables = c(all_of(immune.age.markers),IMMAX)
  ) |>
  modify_column_hide(c(stat_1,stat_2)) %>%
  modify_footnote_header("T.gondii+ effect as standardized mean difference adjusted
for sex and age by ANCOVA",
                        columns = c(Delta_95CI_adj,p.value)) %>%
  modify_header(label ~ "**Characteristics**",p.value = "**P_adj**",
                Delta_95CI_adj="**Delta_adj**\n(95% CI)") %>%
  as_flex_table()
```

**Characteristics**	**Delta_adj** (95% CI)[1]	**P_adj**[1]
Immunosenescence biomarkers		
IMMAX	-0.02 (-0.17, 0.14)	0.823
log.NK.T	0.04 (-0.14, 0.22)	0.676
log.T.CD4pos.CD8pos	0.01 (-0.16, 0.18)	0.928
log.T.CD4.mem.naive	-0.03 (-0.20, 0.14)	0.730
log.T.CD8.mem.naive	-0.06 (-0.21, 0.10)	0.460
logit.CD8posCD28neg.T.cells	0.05 (-0.13, 0.22)	0.600

[1]T.gondii+ effect as standardized mean difference adjusted for sex and age by ANCOVA

```r
# Regression models from Fig. 5 with different treatment of
# intermediate IgG level marked as '0.5' in dataset
IMMAX.regression.intermediate.seropositive=DVS.dat %>%
  mutate(
    T.gondii=factor(ifelse(T.gondii.IgG==0.5,1,T.gondii.IgG),
                    levels = c(0,1),
                    labels = c("neg","pos"))
  ) %>%
  na.omit() %>%
  select(T.gondii,sex,age,IMMAX) %>%
  lm(scale(IMMAX)~T.gondii+scale(age)+sex
     + sex:scale(age)
     + T.gondii:scale(age),
     .)
#
IMMAX.regression.intermediate.seronegative=DVS.dat %>%
  mutate(
    T.gondii=factor(ifelse(T.gondii.IgG==0.5,0,T.gondii.IgG),
                    levels = c(0,1),
                    labels = c("neg","pos"))
  ) %>%
  na.omit() %>%
  select(T.gondii,sex,age,IMMAX) %>%
  lm(scale(IMMAX)~T.gondii+scale(age)+sex
     + sex:scale(age)
     + T.gondii:scale(age),
     .)
#
IMMAX.regression.intermediate.left.out=DVS.dat %>%
  mutate(
    T.gondii=factor(ifelse(T.gondii.IgG==0.5,NA,T.gondii.IgG),
                    levels = c(0,1),
                    labels = c("neg","pos"))
  ) %>%
  na.omit() %>%
  select(T.gondii,sex,age,IMMAX) %>%
  lm(scale(IMMAX)~T.gondii+scale(age)+sex
     + sex:scale(age)
     + T.gondii:scale(age),
     .)
```

```
# plot models as in Fig. 5
models=list("M1"=IMMAX.regression.intermediate.seropositive,
            "M2"=IMMAX.regression.intermediate.seronegative,
            "M3"=IMMAX.regression.intermediate.left.out)
t1=ggcoef_compare(models,
                  type = "faceted",
                  show_p_values = F,
                  interaction_sep = " x ",
                  signif_stars = T,
                  add_reference_rows = F,
                  colour = NULL,
                  facet_row=NULL,
                  return_data = TRUE)
t1=t1 %>%
  mutate(
    model1=factor(case_when(
      model=="M1" ~ "intermediate to pos",
      model=="M2" ~ "intermediate to neg",
      model=="M3" ~ "intermediate to NA"),
      levels = c("intermediate to pos","intermediate to neg","intermediate to NA")
    ),
    label1=case_when(
      label=="scale(age)" ~ "Age\n(SD units)",
      label=="male" ~ "Sex\n(male vs female)",
      label=="pos" ~ "T.gondii\n(pos vs neg)",
      label=="scale(age) x male" ~ "Sex x Age",
      label=="pos x scale(age)" ~ "T.gondii x Age"#,
    ),
    label=label1
  )
#
cowplot.size=9
label_size=cowplot.size-1
ggcoef_plot(
  t1,
  point_stroke = 0.5)+
  labs(x="Standardized regression coefficients with 95% CI",
       caption ="Comparison of models from Fig.5 predicting IMMAX
       with different treatment of the intermediate IgG antibody level (33 IU/ml)
       between the lower (30 IU/ml) and upper cut-offs (35 IU/ml)") +
  facet_wrap(vars(model1),ncol=3,scales="fixed") +
  scale_shape_manual(labels = c("P<0.05", "P>0.05"),values = c(19,21)) +
  theme_minimal(cowplot.size) +
  theme(
    strip.background = element_blank(),
    legend.position = "top",
    axis.ticks.x = element_line(color="black"),
    axis.ticks.length.x = unit(2,"pt"),
    strip.text = element_text(size=label_size),
    panel.grid = element_blank(),
    panel.border = element_rect(fill=NA,color = "black",
                                linewidth = 0.5, linetype = 1)
  )
```

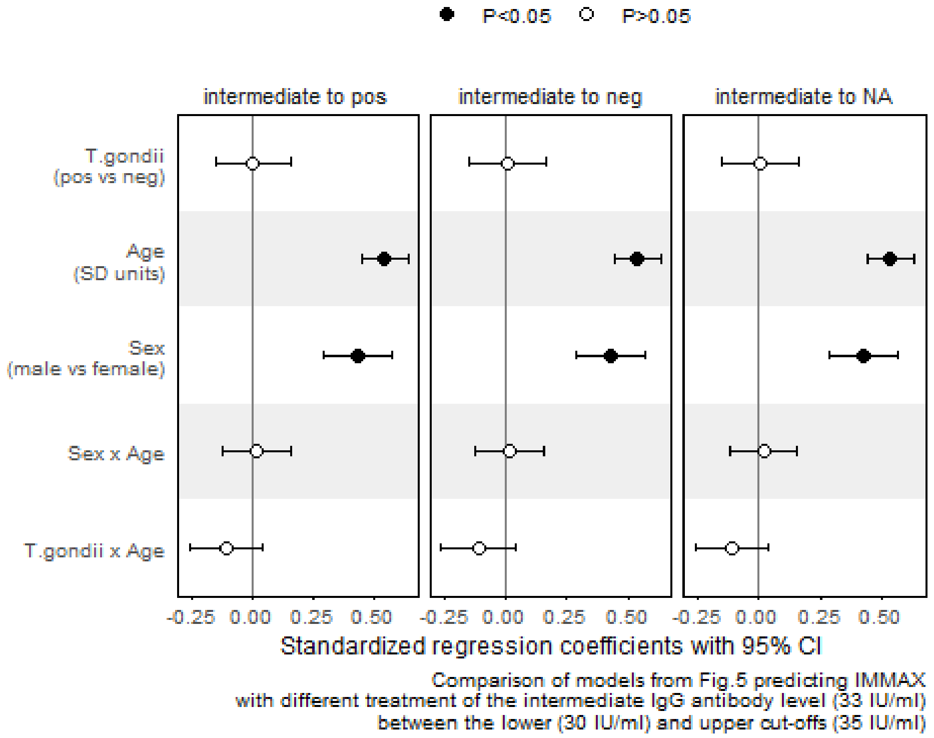
P<0.05
P>0.05
intermediate to pos
intermediate to neg
intermediate to NA
T.gondii (pos vs neg)
Age (SD units)
Sex (male vs female)
Sex x Age
T.gondii x Age
-0.25 0.00 0.25 0.50
Standardized regression coefficients with 95% CI
Comparison of models from Fig.5 predicting IMMAX
with different treatment of the intermediate IgG antibody level (33 IU/ml)
between the lower (30 IU/ml) and upper cut-offs (35 IU/ml)

```
# Information on parameter representing interaction of T.gondii status with age
sprintf("Standardized interaction effect of change in IMMAX with age in SD units
(95%%-CI): %1$4.3f (%2$4.3f to %3$4.3f)",
        coef(IMMAX.regression.intermediate.seropositive)["T.gondiipos:scale(age)"],

confint(IMMAX.regression.intermediate.seropositive)["T.gondiipos:scale(age)",1],

confint(IMMAX.regression.intermediate.seropositive)["T.gondiipos:scale(age)",2]
)

## [1] "Standardized interaction effect of change in IMMAX with age in SD units (95%-
CI): -0.105 (-0.256 to 0.045)"

sprintf("Change in IMMAX with age expressed in decades (95%%-CI): %1$4.3f (%2$4.3f to
%3$4.3f)",
        coef(IMMAX.regression.intermediate.seropositive)["T.gondiipos:scale(age)"]*
          sd(DVS.dat$IMMAX) / sd(DVS.dat$age) *10,

confint(IMMAX.regression.intermediate.seropositive)["T.gondiipos:scale(age)",1]*
          sd(DVS.dat$IMMAX) / sd(DVS.dat$age) *10,

confint(IMMAX.regression.intermediate.seropositive)["T.gondiipos:scale(age)",2]*
          sd(DVS.dat$IMMAX) / sd(DVS.dat$age) *10)

## [1] "Change in IMMAX with age expressed in decades (95%-CI): -0.009 (-0.022 to
0.004)"
```

```
# Posthoc power for observed interaction
#
sprintf("Observed posthoc power for the interaction of age with T.gondii status is
%.3f",
        power.t.regression(
          beta =
coef(IMMAX.regression.intermediate.seropositive)["T.gondiipos:scale(age)"], #
estimated predictor
          k.total = summary(IMMAX.regression.intermediate.seropositive)$df[1], #
total number of predictors
          r.squared = summary(IMMAX.regression.intermediate.seropositive)$r.squared,
# observed R.squared
          n = summary(IMMAX.regression.intermediate.seropositive)$df[1]+
            summary(IMMAX.regression.intermediate.seropositive)$df[2],  # number of
observations
          alpha = 0.05, # type-1 error
          alternative = "two.sided",
          verbose=F)$power)

## [1] "Observed posthoc power for the interaction of age with T.gondii status is
0.875"
```

```
# Regression diagnostics for linear model from Fig. 5 predicting IMMAX
p1 <- function() {
  par(mfrow = c(2, 2), oma = c(0, 0, 2, 0), cex=0.5)
  plot(IMMAX.regression.intermediate.seropositive,which=1:4,sub.caption="Diagnostic
plots")
}
#
ggdraw(p1) +
  theme(plot.background = element_rect(fill = "white"))
```

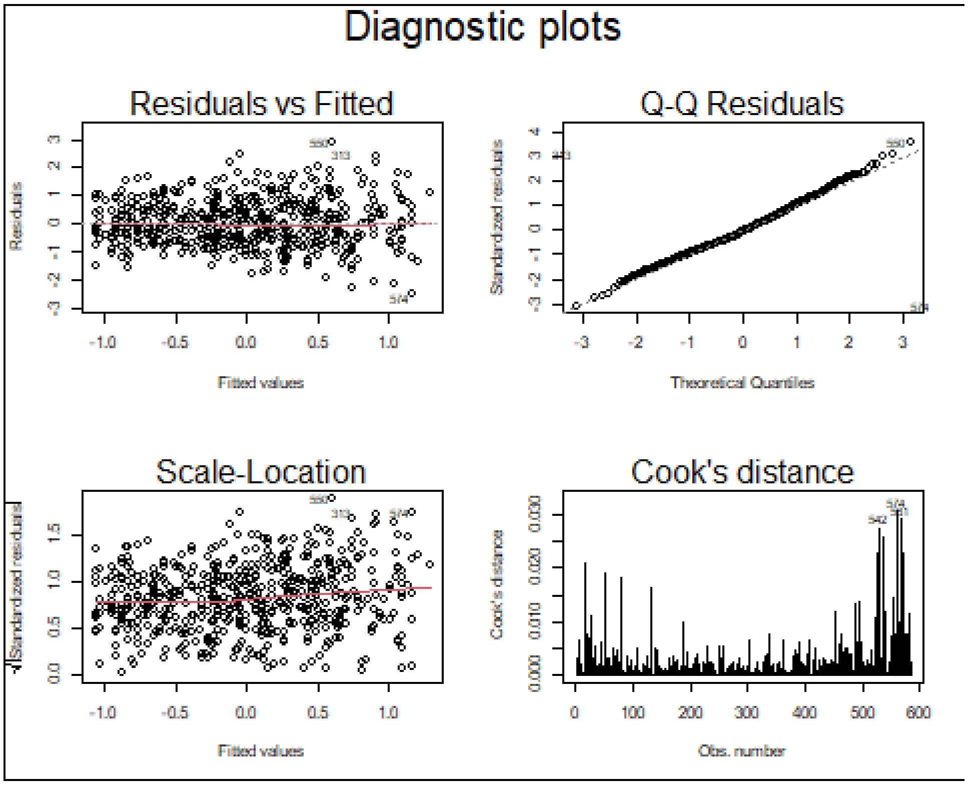

```r
# Considering potential non-linear age effect by spline regression
# fitted using natural cubic splines for age
# default is equivalent to linear regression model
IMMAX.spline.regression.intermediate.seropositive.linear=DVS.dat %>%
  mutate(
    T.gondii=factor(ifelse(T.gondii.IgG==0.5,1,T.gondii.IgG),
                    levels = c(0,1),
                    labels = c("neg","pos")),
    IMMAX=scale(IMMAX),
    age=scale(age)
  ) %>%
  na.omit() %>%
  select(T.gondii,sex,age,IMMAX) %>%
  lm(IMMAX~T.gondii+sex+ns(age)
     + sex:ns(age)
     + T.gondii:ns(age),
     .)
df=4 # regression with natural cubic splines with two interior knots for age effect
IMMAX.spline.regression.intermediate.seropositive.4=DVS.dat %>%
  mutate(
    T.gondii=factor(ifelse(T.gondii.IgG==0.5,1,T.gondii.IgG),
                    levels = c(0,1),
                    labels = c("neg","pos")),
    IMMAX=scale(IMMAX),
    age=scale(age)
  ) %>%
  na.omit() %>%
  select(T.gondii,sex,age,IMMAX) %>%
  lm(IMMAX~T.gondii+sex+ns(age,df=df,intercept = T)
     + sex:ns(age,df=df,intercept = T)
     + T.gondii:ns(age,df=df,intercept = T),
     .)
# parameter lists
attr(terms(IMMAX.spline.regression.intermediate.seropositive.linear), "predvars")

## list(IMMAX, T.gondii, sex, ns(age, knots = numeric(0), Boundary.knots = c(-
1.69399470414971,
## 1.81936884163988), intercept = FALSE))

attr(terms(IMMAX.spline.regression.intermediate.seropositive.4), "predvars")

## list(IMMAX, T.gondii, sex, ns(age, knots = c(-0.639985640412837,
## 0.554557965155624), Boundary.knots = c(-1.69399470414971, 1.81936884163988
## ), intercept = TRUE))

# spline regression model not significantly superior to linear model
anova(IMMAX.spline.regression.intermediate.seropositive.linear,
      IMMAX.spline.regression.intermediate.seropositive.4)

## Analysis of Variance Table
##
## Model 1: IMMAX ~ T.gondii + sex + ns(age) + sex:ns(age) + T.gondii:ns(age)
## Model 2: IMMAX ~ T.gondii + sex + ns(age, df = df, intercept = T) + sex:ns(age,
##     df = df, intercept = T) + T.gondii:ns(age, df = df, intercept = T)
##   Res.Df    RSS Df Sum of Sq      F Pr(>F)
## 1    578 389.13
## 2    572 387.75  6    1.3772 0.3386 0.9165
```

```r
# Difference plot for model fitted as generalized additive model (GAM)
DVS.dat %>%
  mutate(
    T.gondii=factor(ifelse(T.gondii.IgG==0.5,1,T.gondii.IgG),
                    levels = c(0,1),
                    labels = c("neg","pos"))
  ) %>%
  na.omit() %>%
  select(T.gondii,sex,age,IMMAX) %>%
  gam(IMMAX~T.gondii+sex+s(age)
      + s(age,by=sex)
      + s(age,by=T.gondii),
      gaussian(),
      .)  %>%
  get_difference("age",
                 list(T.gondii = c("neg","pos"))) %>%
  plot() +
  labs(x="Age (years)",
       caption =
         "Plot of the non-significant interaction getween age and T.gondii status
       as differences between seropositive and -negative groups
       related to age with 95%-confidence band
       considering potential non-linear age effect by spline regression
       fitted as generalized additive model (GAM)") +
  theme_classic()
```

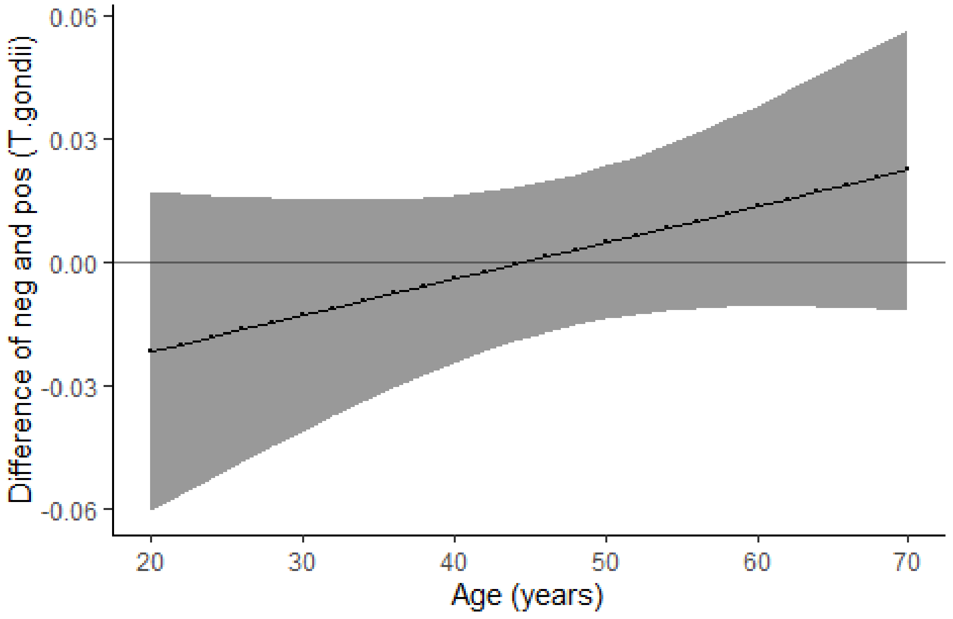

Plot of the non-significant interaction getween age and T.gondii status
as differences between seropositive and -negative groups
related to age with 95%-confidence band
considering potential non-linear age effect by spline regression
fitted as generalized additive model (GAM)

```
# Directed acyclic graph (DAG) illustrating future studies including CMV effect
#
dag.seed=2919
dagify(
  IMMAX ~ Age + Sex + CMV,
  T.gondii ~Age,
  CMV ~  Age,
  CMV~~T.gondii
) %>%
  ggdag(node_size = 22,stylized = F,seed=dag.seed) +
  labs(caption="Directed acyclic graph (DAG) illustrating future studies including
CMV effects") +
  theme_dag_blank()
```

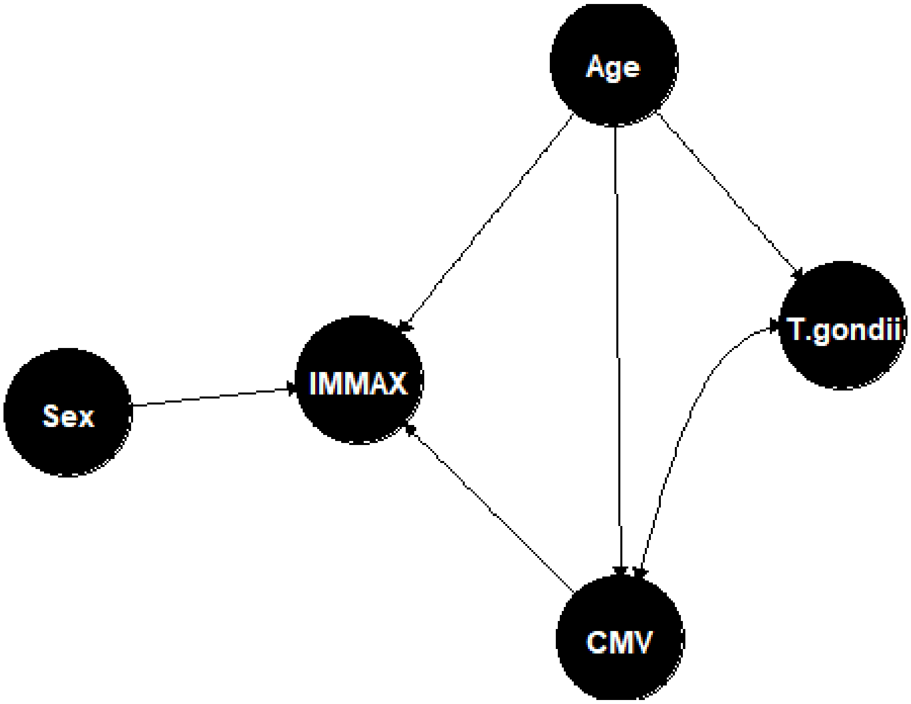

Directed acyclic graph (DAG) illustrating future studies including CMV effects

```r
# citations of used packages
sapply(packages, function(x) report::clean_citation(citation(package=x)))

cowplot
Wilke C (2024). _cowplot: Streamlined Plot Theme and Plot Annotations for 'ggplot2'_.
doi:10.32614/CRAN.package.cowplot <https://doi.org/10.32614/CRAN.package.cowplot>, R
package version 1.1.3, <https://CRAN.R-project.org/package=cowplot>.

ggdag
Barrett M (2024). _ggdag: Analyze and Create Elegant Directed Acyclic Graphs_.
doi:10.32614/CRAN.package.ggdag <https://doi.org/10.32614/CRAN.package.ggdag>, R
package version 0.2.13, <https://CRAN.R-project.org/package=ggdag>.

ggstats
Larmarange J (2025). _ggstats: Extension to 'ggplot2' for Plotting Stats_.
doi:10.32614/CRAN.package.ggstats <https://doi.org/10.32614/CRAN.package.ggstats>, R
package version 0.9.0, <https://CRAN.R-project.org/package=ggstats>.

gridGraphics
Murrell P, Wen Z (2020). _gridGraphics: Redraw Base Graphics Using 'grid' Graphics_.
doi:10.32614/CRAN.package.gridGraphics
<https://doi.org/10.32614/CRAN.package.gridGraphics>, R package version 0.5-1,
<https://CRAN.R-project.org/package=gridGraphics>.

gtsummary
Sjoberg D, Whiting K, Curry M, Lavery J, Larmarange J (2021). \Reproducible Summary
Tables with the gtsummary Package.\ _The R Journal_, *13*, 570-580. doi:10.32614/RJ-
2021-053 <https://doi.org/10.32614/RJ-2021-053>, <https://doi.org/10.32614/RJ-2021-
053>.

mgcv
Wood SN (2011). \Fast stable restricted maximum likelihood and marginal likelihood
estimation of semiparametric generalized linear models.\ _Journal of the Royal
Statistical Society (B)_, *73*(1), 3-36. doi:10.1111/j.1467-9868.2010.00749.x
<https://doi.org/10.1111/j.1467-9868.2010.00749.x>.
Wood SN, Pya N, Säfken B (2016). \Smoothing parameter and model selection for general
smooth models (with discussion).\ _Journal of the American Statistical Association_,
*111*, 1548-1575. doi:10.1080/01621459.2016.1180986
<https://doi.org/10.1080/01621459.2016.1180986>.
Wood SN (2004). \Stable and efficient multiple smoothing parameter estimation for
generalized additive models.\ _Journal of the American Statistical Association_,
*99*(467), 673-686. doi:10.1198/016214504000000980
<https://doi.org/10.1198/016214504000000980>.
Wood SN (2017). _Generalized Additive Models: An Introduction with R_, 2 edition.
Chapman and Hall/CRC. Wood SN (2003). \Thin-plate regression splines.\ _Journal of
the Royal Statistical Society (B)_, *65*(1), 95-114. doi:10.1111/1467-9868.00374
<https://doi.org/10.1111/1467-9868.00374>.

pwrss
Bulus M, Jentschke S (2025). _pwrss: Statistical Power and Sample Size Calculation
Tools_. R package version 1.0.0, <https://doi.org/10.32614/CRAN.package.pwrss>.

report
Makowski D, Lüdecke D, Patil I, Thériault R, Ben-Shachar M, Wiernik B (2023).
\Automated Results Reporting as a Practical Tool to Improve Reproducibility and
Methodological Best Practices Adoption.\ _CRAN_. doi:10.32614/CRAN.package.report
```

```
<https://doi.org/10.32614/CRAN.package.report>,
<https://easystats.github.io/report/>.

splines
R Core Team (2025). _R: A Language and Environment for Statistical Computing_. R
Foundation for Statistical Computing, Vienna, Austria. <https://www.R-project.org/>.

tidygam
Coretta S (2024). _tidygam: Tidy Prediction and Plotting of Generalised Additive
Models_. doi:10.32614/CRAN.package.tidygam
<https://doi.org/10.32614/CRAN.package.tidygam>, R package version 1.0.0,
<https://CRAN.R-project.org/package=tidygam>.

tidyverse
Wickham H, Averick M, Bryan J, Chang W, McGowan LD, François R, Grolemund G, Hayes A,
Henry L, Hester J, Kuhn M, Pedersen TL, Miller E, Bache SM, Müller K, Ooms J,
Robinson D, Seidel DP, Spinu V, Takahashi K, Vaughan D, Wilke C, Woo K, Yutani H
(2019). \Welcome to the tidyverse.\ _Journal of Open Source Software_, *4*(43), 1686.
doi:10.21105/joss.01686 <https://doi.org/10.21105/joss.01686>.
```
